# The Effect of Intravenous Tranexamic Acid on Myomectomy: A Systematic Review and Meta-Analysis of Randomized Controlled Trials

**DOI:** 10.3390/jpm12091492

**Published:** 2022-09-13

**Authors:** Nikolaos Kathopoulis, Anastasia Prodromidou, Dimitrios Zacharakis, Ioannis Chatzipapas, Michail Diakosavvas, Konstantinos Kypriotis, Themos Grigoriadis, Athanasios Protopapas

**Affiliations:** 1st Department of Obstetrics and Gynaecology, National and Kapodistrian University of Athens, Alexandra Hospital, Lourou 2-4, 11528 Athens, Greece

**Keywords:** tranexamic acid, myomectomy, benign gynecology, hemostatic agent, meta-analysis

## Abstract

Myomectomy is the preferred surgical treatment for symptomatic women with uterine myomas who wish to preserve their fertility. The procedure may be associated with significant intraoperative blood loss, which predisposes to increased transfusion rates and morbidity. The objective of our systematic review and meta-analysis is to investigate whether intravenous (IV) use of tranexamic acid (TXA) may reduce blood loss during myomectomy. Three electronic databases were screened until June 2022. The eligible studies were assessed for risk of bias. Four randomized controlled trials that reported outcomes from a total of 310 women were finally included in the meta-analysis—155 patients received intravenous TXA while the remaining 155 received placebo injection with normal saline or water for injection. Total estimated blood loss was significantly lower in patients who received TXA before myomectomy compared to control (230 patients MD −227.09 mL 95% CI −426.26, −27.91, *p* = 0.03). This difference in favor of TXA group remained when intraoperative and postoperative blood loss was separately analyzed. Postoperative hematocrit values and hemoglobin levels did not differ among the two groups (180 patients MD 0.67% 95% CI −0.26, 1.59, *p* = 0.16 and 250 patients MD 0.17 mg/dL 95% CI 0.07, 0.41, *p* = 0.17, respectively). The number of patients that received blood transfusion was also not different (310 patients OR 0.46 95% CI −0.14, 1.49, *p* = 0.19). Total operative time was significantly prolonged in control group compared to TXA (310 patients MD −16.39 min 95% CI −31.44, −1.34 *p* = 0.03). Our data show that the IV use of TXA may significantly reduce intraoperative blood loss in patients undergoing myomectomy and contribute to reduced operative time.

## 1. Introduction

Uterine myomas are benign tumors arising from myometrial smooth muscle cells. Leiomyomas are the most common gynecologic tumor while the majority are asymptomatic. However, abnormal uterine bleeding, pelvic pressure and infertility are the most common symptoms and are related to fibroma’s size, number and position [1]. Myomectomy is the preferred surgical treatment option for symptomatic women of reproductive age who wish to preserve their fertility. Depending on myomas characteristics and the surgeon’s experience, laparoscopic, robotic, hysteroscopic or abdominal approaches may be chosen to achieve the best operative outcome. Irrespective of the surgical approach, myomectomy may be associated with significant intraoperative blood loss, which predisposes to increased transfusion rate and significant morbidity [2].

Various interventions are available to reduce blood loss, including pharmacological agents, energy use, tourniquets and barbed sutures. Regarding drug use, intra-myometrial vasopressin injection, vaginal or rectal misoprostol and intravenous oxytocin infusion have been proposed, aiming to reach a positive effect on intraoperative blood loss and, in some cases, on transfusion rates [3,4]. Concerning vasopressin, some safety concerns were why many countries banned its use during myomectomy [5]. Other agents were then applied to facilitate surgeons performing myomectomy safely. Tranexamic acid (TXA) is an antifibrinolytic factor that is widely used in various surgical procedures such as cardiac surgery, trauma, urological and orthopedic operations [6,7,8]. In gynecology, TXA has been claimed to reduce abnormal uterine bleeding and is considered an effective non-hormonal medication in managing menorrhagia [9,10]. Recent augmented literature supports the use of TXA as a hemostatic agent during myomectomy, mainly during open but also in minimally invasive procedures.

The scope of the present review is to evaluate the effectiveness of intravenous (IV) TXA in reducing blood loss during myomectomy (abdominal, laparoscopic, or robotic) and the respective impact on elimination of transfusion rates.

## 2. Materials and Methods

### 2.1. Study Design and Eligibility Criteria

The present meta-analysis was designed according to the updated guidelines for Systematic Reviews and Meta-analyses (PRISMA) based on the authors’ predetermined inclusion criteria [11]. No language restrictions were applied. Randomized controlled trials (RCTs) that evaluated the effect of intravenous TXA administration on perioperative outcomes, compared to placebo treatment in patients who had myomectomy, were assessed and critically appraised. Only comparative RCTs that reported at least one of the predetermined primary outcomes were considered eligible. Non-randomized trials, conference papers, letters to the editor, editorials, case reports, reviews and experimental animal studies were excluded from tabulation and analysis. The study`s protocol was published in Open Science Framework (DOI: 10.17605/OSF.IO/4D83Q)

The inclusion criteria were as follows: studies with a total number of more than 50 cases, female patients aged >18 years and women who underwent open or minimally invasive myomectomy. Studies that included patients who had hysteroscopic myomectomy were excluded. Additionally, the use of agents other than saline and water for injection or no injection at all as control group was also considered a criterion for exclusion. Topical application of TXA alone or any other combinations of TXA with other hemostatic agents were not included.

### 2.2. Information Sources

The literature search was systematic and was performed in three stages. Initially, 3 electronic databases (PubMed, Scopus and Google Scholar) were searched until June 2022. The date of the last search was 30 June 2022. Titles and/or abstracts of comparative articles that evaluated the perioperative outcomes of patients who received intravenous TXA injection prior to myomectomy were assessed for eligibility. Studies that were deemed to meet criteria were retrieved in full text. Finally, the references of the eligible articles were also searched for further relevant studies in the field. A minimum number of search keywords were utilized in an attempt to assess a number of studies that could be easily searched while simultaneously minimizing the potential loss of articles. The following key words were utilized: “tranexamic acid”, “myomectomy”, “fibroid”, “open myomectomy” and “laparoscopic myomectomy”.

### 2.3. Search

The search was performed using keywords and Boolean operators. Our search strategy in PubMed used the following search terms:

- (“tranexamic acid” [MeSH Terms] OR (“tranexamic” [All Fields] AND “acid” [All Fields]) OR “tranexamic acid” [All Fields]) AND (“uterine myomectomy” [MeSH Terms] OR (“uterine” [All Fields] AND “myomectomy” [All Fields]) OR “uterine myomectomy” [All Fields] OR “myomectomies” [All Fields] OR “myomectomy” [All Fields])
- (“tranexamic acid” [MeSH Terms] OR (“tranexamic” [All Fields] AND “acid” [All Fields]) OR “tranexamic acid” [All Fields]) AND (“fibroid s” [All Fields] OR “leiomyoma” [MeSH Terms] OR “leiomyoma” [All Fields] OR “fibroid” [All Fields] OR “fibroids” [All Fields])

The PICO criteria that were used to develop our search strategy were as follows:

Patient/Problem: Female adult patients with fibroids, Intervention: Myomectomy, Comparison: Tranexamic acid versus placebo control, Outcome: blood loss, blood transfusion, hemoglobin levels, hematocrit values.

### 2.4. Selection of Sources of Evidence

**Figure 1:**
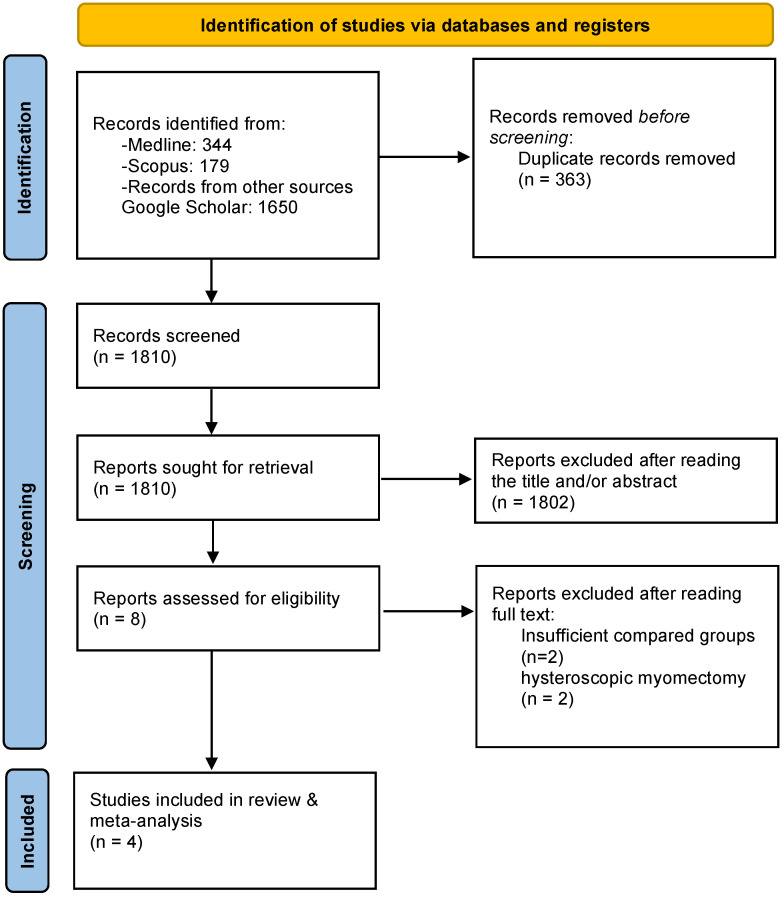
Search flow diagram.

The studies were initially selected based on their title and then on the abstract in case of ambiguity for eligibility of the study. After exclusion of duplicates, the predetermined inclusion and exclusion criteria were applied. Articles that fulfilled or were deemed to fulfil the inclusion criteria were retrieved. Three authors (AP, NK and DZ) performed an independent and meticulous search of the literature, excluded overlaps and tabulated the selected indices in structured form. Discrepancy among the authors was discussed by all of them until they reached a consensus. The PRISMA flow diagram schematically presents the stages of article selection (Figure 1).

### 2.5. Data-Charting Process, Data Items and Synthesis of Results

The variables to be extracted by the included studies were discussed by all authors as structured tables to be independently fulfilled by two of them (AP and NK). After extraction, all authors discussed the validity of the extracted data and resolved potential discrepancies to achieve accuracy and validity. Data on patients characteristics included patients’ age, preoperative hematocrit and hemoglobin, number, weight and size of fibroids, type of surgical repair and TXA administration aspects. Our primary outcomes were estimated blood loss (EBL) (total, intraoperative and postoperative), postoperative hemoglobin levels and postoperative hematocrit values. Our secondary outcomes were operative time (OT) and length of hospital stay (LOS).

### 2.6. Quality Assessment

**Figure 2:**
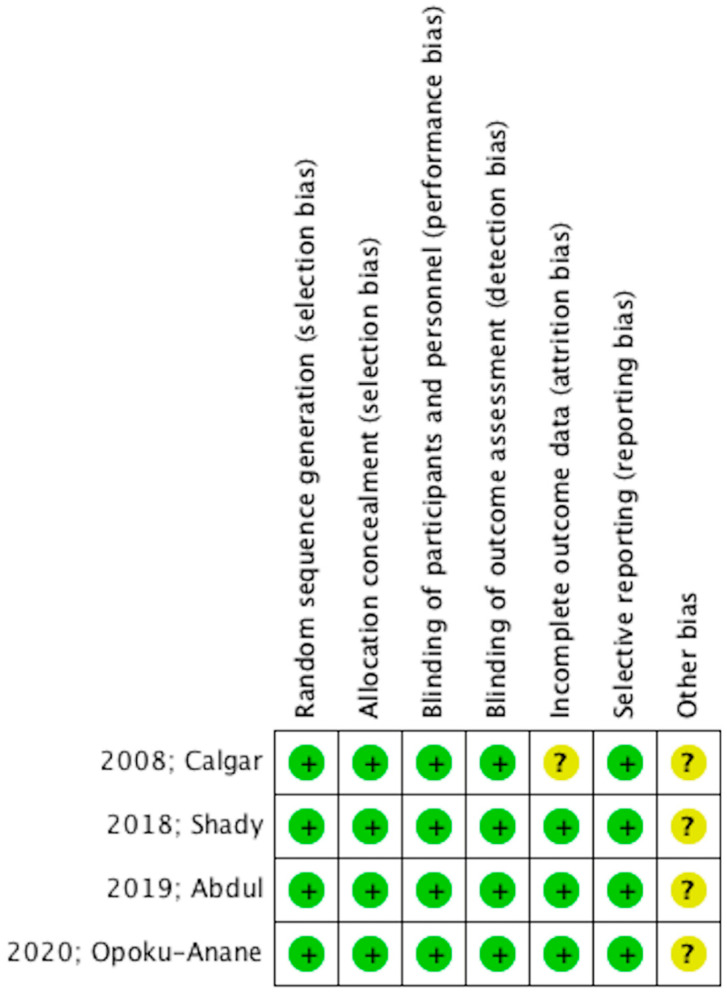
RCT methodological quality. Risk-of-bias summary: “?” represents unclear bias, and “+” represents low risk of bias.

The evaluation of the methodological quality of the included studies was made using the Review Manager 5.4 tool for the assessment of the “Risk of Bias”, according to Cochrane Collaboration Handbook (Figure 2) [12]. Two authors (NK and AP) independently performed the procedure.

### 2.7. Statistical Analysis

Statistical meta-analysis was performed using the RevMan 5.4 software (Copenhagen: The Nordic Cochrane Centre, The Cochrane Collaboration, 2011). Confidence intervals (CI) were set at 95%. Mean difference (MD) and odds ratios (OR) were used in the analysis. The results were calculated using the DerSimonian-Laird random effect model (REM) revealing significant heterogeneity in the methodological characteristics of the included studies [13]. The significant heterogeneity of the included studies, which may influence the methodological integrity of the tests and their limited number (*n* < 10 studies), precluded the test of publication bias. Mean values and standard deviations were calculated according to the equations proposed by Hozo et al., Luo et al. and Wan et al. when not provided by the studies [14,15,16]. The cut-off for statistical significance was set at *p* < 0.05.

## 3. Results

### 3.1. Excluded Studies

A total of four studies were excluded from tabulation and analysis [17,18,19,20]. More specifically, in the study by Ngichabe et al. all patients received ornipressin with TXA as adjunct hemostatic in one group and was thus excluded [17]. The studies by Rasheedy et al. and Mousa et al. were also not included as the myomectomy was performed through hysteroscopic approach [18,19]. Finally, another study was excluded due to an insufficient control group [20].

### 3.2. Included Studies

Four RCTs that reported outcomes of a total of 310 women were finally included in the meta-analysis [21,22,23,24]. Among them, 155 patients received intravenous TXA before myomectomy while the remaining 155 received placebo injection with normal saline or water for injection before myomectomy. The analyzed indices were tabulated in two structured tables. Table 1 depicts the main characteristics of the included studies and patients.

### 3.3. Quality Assessment

The assessment of the methodological quality of the included studies according to the “Risk of Bias” assessment tool is depicted in Figure 2. In particular, the selection bias was low as all of the studies were randomized and double-blinded, which further limits performance bias. Detection and attrition bias were low while reporting bias was moderate. Therefore, the aforementioned parameters provide a generally good study quality.

### 3.4. Patient Characteristics

Neither the mean number of resected fibroids nor the total mean weight of these were different among the two groups (210 patients MD −0.04 fibroids 95% CI −0.82, 0.74, *p* = 0.92 and 140 patients MD 31.27 gr 95% CI −170.32, 232.85, *p* = 0.76, respectively). The size of the larger fibroid was also not different (130 patients MD −0.18 cm 95% CI −1.30, 0.95, *p* = 0.76). Preoperative hemoglobin and hematocrit levels did not differ among the two groups (310 patients MD 0.3 mg/dL 95% CI −0.46, 1.13, *p* = 0.40 and 240 patients MD −0.06% 95% CI −1.03, 0.90, *p* = 0.90, respectively).

### 3.5. Main Outcomes

**Figure 3:**
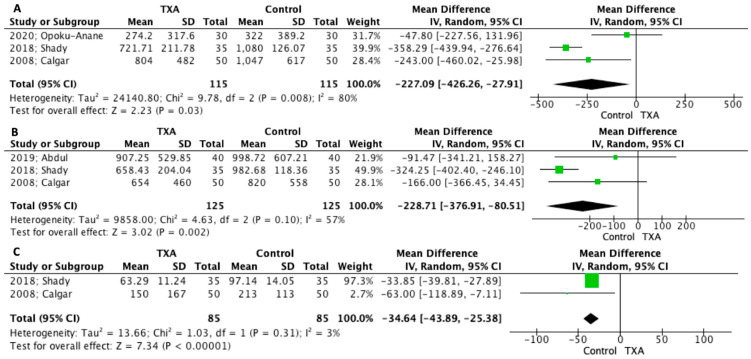
Forest plot depicting estimated blood loss (EBL) (**A**) total EBL, (**B**) intraoperative EBL and (**C**) postoperative EBL [21,22,23,24].

Table 2 depicts the main outcomes as reported from the included studies. Total estimated blood loss was significantly lower in patients who received TXA before myomectomy compared to control (230 patients MD −227.09 mL 95% CI −426.26, −27.91, *p* = 0.03) (Figure 3a). This difference in favor of TXA group remained when intraoperative and postoperative blood loss was separately analyzed (250 patients MD −228.71 95% CI −376.91, −80.51, *p* = 0.002 and 170 patients MD −34.64 mL 95% CI −43.89, −25.38, *p* < 0.00001, respectively) (Figure 3b,c). Postoperative hematocrit values did not differ among the two groups (180 patients MD 0.67% 95% CI −0.26, 1.59, *p* = 0.16). The same was also detected after the analysis of hemoglobin levels (250 patients MD 0.17 mg/dL 95% CI 0.07, 0.41, *p* = 0.17).

**Figure 4:**
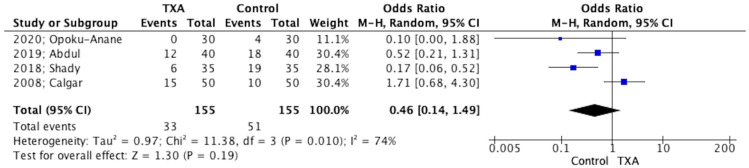
Forest plot depicting blood transfusion [21,22,23,24].

The number of patients receiving blood transfusion was not different among patients who received TXA before myomectomy versus those who received myomectomy alone (310 patients OR 0.46 95% CI −0.14, 1.49, *p* = 0.19) (Figure 4). Similarly, among the patients that received blood transfusion, the mean number of units transfused in each patient was comparable among the two groups (180 patients OR −0.08 units 95% CI −0.38, 0.22, *p* = 0.61).

### 3.6. Secondary Outcomes

**Figure 5:**
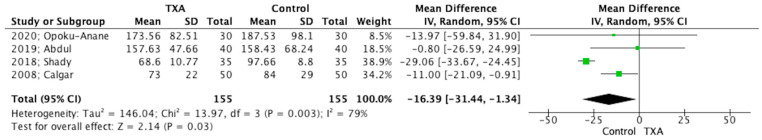
Forest plot depicting operative time [21,22,23,24].

Total operative time was significantly prolonged in control group compared to TXA (310 patients MD −16.39 min 95% CI −31.44, −1.34 *p* = 0.03) (Figure 5). This was also observed in subgroup analysis of patients who had open myomectomy (250 patients MD −16.42 min 95% CI −32.87, −0.03 *p* = 0.05). Finally, length of hospital stay did not differ among the two groups (150 patients MD −0.45 days 95% CI −1.07, 0.17, *p* = 0.15).

## 4. Discussion

### 4.1. Summary of Findings

This systematic review and meta-analysis aimed to summarize the efficacy of prophylactic intravenous use of TXA in women who had myomectomy. Four studies, all double blind randomized controlled, comparing IV use of TXA with placebo were included in the analysis with a total of 310 patients (155 patients received intravenous TXA and 155 received placebo). According to the quality assessment, the studies had an overall low risk of bias. The pooled findings revealed a positive effect of TXA use on total estimated blood loss, intraoperative and postoperative blood loss compared with placebo. Moreover, the total operative time was significantly reduced when TXA was used. While using TXA managed to reduce blood loss, there was no positive impact on postoperative hematocrit, hemoglobin change, or transfusion rate.

### 4.2. Interpretation of Results and Clinical Implications

Myomectomy, irrespectively of the surgical route (abdominal, laparoscopic, robotic), is an operation characterized by significant intraoperative blood loss. The blood diffusion in the operating field may compromise visualization and predispose to severe iatrogenic injuries, related to fibroma number, size and location. Especially during laparoscopic myomectomy, the need to maintain a clean surgical field is one of the principal surgeon’s goals. TXA is an agent widely used lately to reduce intraoperative blood loss. Our findings show that the IV use of TXA helps reduce intraoperative blood loss. We chose strict selection criteria to reduce heterogeneity, so we focused on the IV use of TXA. As shown by our study, the reduction of intraoperative bleeding may keep a favorable surgical field and reduce operating time. Moreover, we can claim that a better visualization permits the surgeon to stay focused on the key surgical steps, minimizing fatigue levels, a factor predisposing to complications.

Although the blood loss was significantly reduced, the transfusion rate and the hemoglobin change were not improved with TXA. This result limits the clinical significance of TXA, but further studies with more patients are needed to ensure safer conclusions.

### 4.3. Comparison with Previous Meta-Analysis

The first meta-analysis conducted in the field is the study of Topsoee et al. in 2017 that searched the effect of TXA in major benign uterine surgery [25]. In this study, 14 out of 16 trials described the effect of TXA during cesarean section, and only in two studies was an abdominal myomectomy performed. One of the two studies was an abstract with no information about the study methods. Therefore, the results should be interpreted with caution. The following comes from Fusca et al. in 2019, where four studies were included in the meta-analysis [26]. One of these studies was not blinded as no intervention was used in the control group, and the other was a study in which hysteroscopic myomectomy was performed. The most recent and complete study was the one conducted by Baradwan et al. in 2022 [27]. In this well-organized meta-analysis, seven RCTs were included in the analysis. Two of the studies, though, had a topical application of the TXA, and in one hysteroscopic myomectomy was the procedure performed. Moreover, one of the referred studies had no intervention in the control group, so no blinding was established, predisposing to performance bias. In our opinion, hysteroscopic myomectomy has a different surgical field than abdominal or laparoscopic and any intervention should be analyzed separately. Unique hysteroscopic technical parameters such as fluid distention media used, intrauterine pressure and fluid absorption may significantly affect the blood loss and operating time or termination to such an extent that it cannot be compared with any other myomectomy surgical route. Moreover, we chose to induce strict selection criteria even if our study had fewer patients in the final analysis. Including only RCTs comparing IV use of TXA with placebo in the control group renders our study more homogenous and the results more reliable. On this basis, our results are consistent with the studies mentioned above regarding the positive effect of TXA use on total, intraoperative and postoperative blood loss, with our study having the advantage of including studies with less heterogeneity.

### 4.4. Strengths and Limitations

We set aside data restrictions to eliminate data losses while three authors independently searched the literature. Additionally, we also aimed to set strict selection criteria to reduce the significant heterogeneity among the studies so as to eliminate the potential bias in the interpretation of the outcomes. This is to our knowledge, the first study that evaluated the effect of intravenous tranexamic acid compared to placebo treatment on perioperative outcomes in patients that had myomectomy based on the existing RCTs.

However, several inherent limitations need to be addressed. The number of eligible studies is limited as well as the number of the included patients, which precludes reaching safe conclusions. Consequently, the potential effect of TXA on the improvement of hemorrhage during myomectomy may be over- or underestimated. Despite the plethora of published and recruited studies comparing TXA with other agents such as dinoprostone, misoprostol and oxytocin, the available outcomes on the comparison with placebo are still limited. However, we have identified other recruited potential eligible RCTs (NCT04560465 and NCT02620748) on the use TXA compared to placebo control. Nonetheless, their outcomes are not yet published, and could probably influence our outcomes. Finally, while laparoscopic myomectomy could benefit more from a clean surgical field due to reduced intraoperative blood loss, there is a lack of evidence. In all the included studies, an abdominal myomectomy was performed except for one study where only a small portion of the sample underwent laparoscopic or robotic myomectomy. It is unknown whether the positive effect of TXA on estimated blood loss would be preserved if the sample was more balanced, including more patients with laparoscopic myomectomy.

## 5. Conclusions

The IV use of TXA may significantly reduce intraoperative blood loss in patients undergoing myomectomy. Moreover, this agent may predispose to reduced operating time in this group of patients. However, more RCTs are necessary to strengthen the evidence of this systematic review and meta-analysis.

## Figures and Tables

**Table 1:** Main characteristics of the included studies.

Year; Author	Country	Type of Study	Inclusion Criteria	Surgical Approach	Compared Groups
2019; Abdul	Nigeria	DB-RCT	No pregnancy; symptomatic fibroid scheduled for abdominal myomectomy, Uterine size <= 28 weeks, women without pre-operative Anemia (i.e., Hemoglobin concentration ≥ 10 g/dL); no current therapy with gonadotropin-releasing hormone analogues, mefenamic acid and other hormones, no previous abdominal surgery, possibility to use hemostatic tourniquet, no chronic liver diseases, no nephropathies, no bleeding disorders and past thromboembolic disorders, no hypersensitivity to TXA, agree to consent	Abdominal myomectomy	TXA (10 mg/kg) 10–15 min before incision + tourniquet tying (cervico-isthmic junction IO) vs. placebo (water for injection) + tourniquet
2020; Opoku-Anane	USA	DB-RCT	Age between 18 & 50 years, fibroids ≥ 10 cm, intramural or broad ligament fibroid ≥6 cm ≥5 fibroids, symptomatic fibroids, uterine sparing surgery, no contraindication to TXA (thromboembolic disease, ischemic heart disease, malignancy, hematuria, liver disease, chronic kidney disease, subarachnoid hemorrhage, no pregnancy, no hypersensitivity to TXA, no use of factor IX complex concentrates, anti- inhibitor coagulant concentrates, and all-trans retinoic acid—within 2 weeks of the planned surgery	Laparoscopic, Robotic or abdominal myomectomy	TXA 15 mg/kg 20 min before incision vs. placebo (normal saline iv bolus 20 min before incision
2018; Shady	Egypt	DB-RCT	Symptomatic leiomyomas, scheduled for abdominal myomectomy with myoma staging from (3 to 6), no vaginal or laparoscopic myomectomy, no preoperative embolization or gonadotrophin releasing hormone analogue, no cervical and broad ligament myoma, no cardiac, hepatic, renal or thromboembolic disease, no allergy to TXA	Abdominal myomectomy	TXA 1 gr TXA (2 amp kapron 500 mg 5 mL iv before skin incision + topical application of normal saline on myoma bed vs. placebo (110 mL normal saline iv before incision) + topical application of normal saline on myoma bed
2008; Calgar	Turkey	DB-RCT	No malignancy, no history of thromboembolic disease, no ischemic heart disease, no sub-arachnoidal bleeding, no hematuria, BMI ≤ 30	Abdominal myomectomy	TXA 10 mg/kg (max 1 g) for 10 min 15 min before incision + continuous infusion of 1 mg/kg/h in 1 L saline for 10 h vs. placebo saline bolus in 15 min before incision + continuous 1 L saline during 10 h

TXA: Tranexamic Acid; DB-RCT: Double blind Randomized controlled trial; PS: Prospective.

**Table 2:** Main outcomes of the included studies.

Year; Author	Patient No	Operative Time (min)	IO Blood Loss (mL)	PO Blood Loss (mL)	PrO/PO Hemoglobin (g/dL)	Number of Fibroids/Fibroid Weight/Size	PrO/PO Hematocrit	Blood Transfusion N (%)	Hospital Stay (Days)
2019; Abdul	40 vs. 40	157.63 ± 47.66 vs. 158.43 ± 68.24	907.25 ± 529.85 vs. 998.72 ± 607.21	N/A	11.40 ± 0.94 vs. 11.34 ± 1.28/10.17 ± 0.73 vs. 10.06 ± 1.18	14.88 ± 11.8 vs. 14.73 ± 13.31/(vol-mls) 941.75 ± 673.59 vs. 778.25 ± 609.3/1027.4 ± 750.45 vs. 845.72 ± 684.33 (g)	34.3 ± 2.4 vs. 34.18 ± 3.47/30.58 ± 2.11 vs. 30.15 ± 3.29	12(30) vs. 18 (30) UNITS 0.75 ± 1.28 vs. 1.13 ± 1.64	4.8 ± 0.61 vs. 5.55 ± 0.71
2020; Opoku-Anane	30 vs. 30	173.56 ± 82.51 vs. 187.53 ± 98.1	200 (100–508) vs. 240 (105–605) (IQR) TOTAL 274.152 ± 317.5954 vs. 321.995 vs. 389.21	N/A	13.39 ± 1.95 vs. 11.11 ± 2.02	6.07 ± 5.45 vs. 6.71 ± 6.23 /324 (178–562) vs. 398 (143–647) 356.8 ± 298.91 vs. 395.86 ± 392.32/8.6 (6–10) vs. 8.5 (7–10) 8.17 ± 3.11 vs. 8.5 ± 2.34	37.8 ± 4.20 vs. 36.78 ± 5.22	None: 30 vs. 26 1 unit: 0 vs. 2 units: 0 vs. 1 4 units: 0 vs. 1	N/A
2018; Shady	35 vs. 35	68.6 ± 10.77 vs. 97.66 ± 8.8	658.43 ± 204.04 vs. 982.68 ± 118.36 721.71 ± 211.78 vs. 1080 ± 126.07 (TOTAL)	63.29 ± 11.24 vs. 97.14 ± 14.05	10.57 ± 0.81 vs. 10.56 ± 0.77/10.02 ± 0.81 vs. 9.83 ± 0.63	4(1–8) vs. 4 (1–8) 4 ± 1.75 vs. 4 ± 1.75/N/A/12.77 ± 4.12 vs. 12.66 ± 3.96	N/A	6 (17.1) vs. 19 (54.3)	3.54 ± 0.85 vs. 3.66 ± 0.84
2008; Calgar	50 vs. 50	73 ± 22 vs. 84 ± 29	654 ± 460 vs. 820 ± 558 804 ± 482 vs. 1047 ± 617 (TOTAL)	150 ± 167 vs. 213 ± 113	11.4 ± 2 vs. 12 ± 1.6/9.97 ± 1.5 vs. 9.76 ± 1.4	Vol (cm^3^) 457 ± 669 vs. 286 ± 259	36 ± 5 vs. 37 ± 4/31.7 ± 3.9 vs. 30.7 ± 3.4	15(30) vs. 10 (20) UNITS 0.3 ± 0.8 vs. 0.3 ± 0.7	N/A

Continuous data is reported in mean ± SD, PrO: Preoperative; IO: intraoperative; PO: postoperative.

## Data Availability

Not applicable.
